# Emerging approaches for preventing cytokine release syndrome in CAR-T cell therapy

**DOI:** 10.1039/d2tb00592a

**Published:** 2022-07-18

**Authors:** Srinivas Balagopal, Koichi Sasaki, Pooja Kaur, Maria Nikolaidi, Jun Ishihara

**Affiliations:** Department of Bioengineering, Imperial College London London W12 0BZ UK k.sasaki@imperial.ac.uk j.ishihara@imperial.ac.uk

## Abstract

Chimeric antigen receptor (CAR) T cells have demonstrated remarkable anti-tumor efficacy against hematological malignancies, such as leukemia and lymphoma. However, patients treated with CAR-T cells frequently experience cytokine release syndrome (CRS), one of the most life-threatening adverse events of the therapy induced by systemic concentrations of pro-inflammatory cytokines throughout the body. Immunosuppressants such as tocilizumab are currently administered to treat the onset and progression of CRS symptoms. In order to reduce the risk of CRS, newly designed next-generation CAR-T treatments are being developed for both hematopoietic malignancies and solid tumors. In this review, we discuss six classes of interesting approaches that control cytokine production of CAR-T cell therapy: adaptor-based strategies, orthogonal cytokine–receptor pairs, regulation of macrophage cytokine activity, autonomous neutralization of key cytokines, kill switches and methods of reversible suppression of CARs. With these strategies, future CAR-T cell therapies will be designed to preemptively inhibit CRS, minimize the patients’ suffering, and maximize the number of benefiting patients.

## Abstract 2

Strategies for reducing CRS in CAR-T cells capitalize on the specificity between CAR-T cells, cytokines, and their receptors, the role of macrophages in cytokine release, and genetic constructs to ablate CAR-T function reversibly and irreversibly.

## Graphical abstract

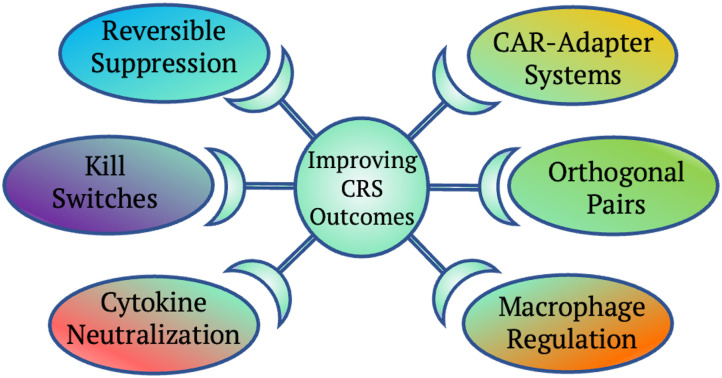

## 1. Introduction

Cancer immunotherapy, exemplified by immune checkpoint inhibitor (CPI) antibodies, which won the Nobel Prize in Physiology or Medicine in 2018, is revolutionizing cancer treatment. The use of anti-programmed cell death-1 (PD-1) and anti-cytotoxic T lymphocyte antigen 4 (CTLA4) antibodies in combination therapy for melanoma patients has achieved prolonged patient survival,^1^ led to approval by the U.S. Food and Drug Administration (FDA). However, patients who literally respond to immunotherapy often experience potent immune-related adverse events (irAEs). In the aforementioned anti-PD-1 and anti-CTLA4 combination, 36% of patients were forced to discontinue treatment due to irAEs. Therefore, reducing irAEs is a pressing issue in cancer immunotherapy, not only to alleviate patient suffering associated with treatment, but also to increase the number of applicable patients. To date, a variety of delivery methods to reduce the side effects of immune-modulating molecules have been developed and well-summarized elsewhere.^2–4^

Chimeric antigen receptor (CAR) T cells, first approved by the FDA in 2017, are another promising cancer immunotherapy modality. Controlling their efficacy and side effects is an equally important issue, but fundamentally different approaches would be possible and required for CAR-T cells, which are living agents, than for non-living therapeutics such as proteins and their development is in its infancy. Therefore, we focus on recent advances in the engineering of adoptive T cell therapy in this review. We first present the basics of CAR-T cells, and then showcase emerging approaches to address cytokine release syndrome (CRS), a life-threatening side effect associated with T cell therapy. Finally, we will discuss the advantages and limitations of the presented methodologies as well as future perspectives.

### 1.1. Chimeric antigen receptor T cells (CAR-T cells)

CAR-T cells are T cells engineered to express a receptor, CAR, that binds to a tumor-associated antigen (TAA) on the surface of tumor cells. Upon antigen-binding, the CAR-T cell becomes activated, causing it to release cytotoxic molecules such as cytokines, perforin and granzymes, which induce apoptosis in the tumor cells. CAR-T cell therapy is a subset of adoptive cell therapy (ACT), which encapsulates treatments that engineer the patient's own T cells *ex vivo* and re-introduce them into the body to eliminate tumor cells.^5^

The CAR has several components: extracellularly, it contains a single-chain variable fragment (scFv), which is derived from an antibody originally targeting the TAA; the scFv binds to the antigen, which induces CAR-T cell activation. The scFv binds to the TAA, thus initiating the CAR-T cell's response, independent of the major histocompatibility complex (MHC); this property presents CARs with an advantage over T cell receptors (TCRs), which are MHC-dependent and prone to tumor cells’ actions to evade detection, such as MHC downregulation.^6,7^ The scFv is connected by a hinge through the transmembrane domain. Within the cell membrane, there is an activation domain and co-stimulatory domains that amplify the CAR signal transduction pathway and increase persistence *in vivo*.^8–10^

Currently, there are five FDA-approved CAR-T cell therapies for treating hematological malignancies, such as acute lymphoblastic leukemia (ALL), diffuse large B-cell lymphoma (DLBCL), and multiple myeloma (MM).^11,12^ Despite their therapeutic success in treating blood-related cancers, CAR-T cells are less effective against solid tumors. Such obstacles include limited T cell trafficking and infiltration within the tumor, a phenomenon induced by a mismatch of chemokine receptors, irregular tumor vasculature,^13^ and immunosuppression (particularly *via* regulatory T cells, among other cell types).^14^ In tandem, these factors render solid tumors as an unfavorable setting for immune cell-mediated anti-tumor activity.

### 1.2. Cytokine release syndrome (CRS) in CAR-T cell therapy

While CAR-T cells’ robustness in the tumor microenvironment (TME) should be augmented, it is also important to consider the safety of the therapy. Most notably, CRS is a frequently-reported adverse side event of CAR-T cell therapy with symptoms of severe fever, organ damage, and hypoxia (among others).^15^ CRS refers to augmented systemic concentrations of pro-inflammatory cytokines, such as interleukin-6 (IL-6), interferon-gamma (IFN-γ), and tumor necrosis factor (TNF), *etc.*^16–19^ The pathophysiology of CRS is usually due to on-target effects – binding of CAR to its target antigen, the initial release of cytokines such as IFN-γ by activated CAR-T cells, and subsequent activation of bystander immune cells – which then results in the release of a huge range of cytokines from both CAR-T cells and endogenous immune cells along with CAR-T expansion.^20^ As described later in this review, among host immune cells, monocytes and macrophages have been found to be the main source of cytokines that are directly linked to severe CRS. CRS is graded on a scale of 1–4 based on the severity of fever, hypotension and hypoxia.^18^ Grade 4 CRS characterizes life-threatening adverse events.^18,21,22^

Tocilizumab, an FDA-approved CRS treatment in CAR-T cell therapy, is a recombinant immunosuppressive monoclonal antibody that binds to the IL-6 membrane and soluble receptors, preventing IL-6 signals. By inhibiting IL-6, which plays a key role in the downstream inflammatory cascade,^23^ tocilizumab has a prominent effect on curtailing CRS without inhibiting CAR-T cell anti-tumor activity.^24–27^ Despite its clinical success, there are unknown factors that surround tocilizumab's use for the future. For blood cancers, there has been no established time for tocilizumab administration that optimizes its efficacy.^28^ Although tocilizumab has been administered to treat severe CRS after it has already become dangerous for the patient,^23^ the effectiveness of preemptive tocilizumab treatment is being tested,^29–31^ indicating a shift towards managing CRS before its effects worsen.^18^ Currently, patients may even experience tocilizumab-refractory CRS, which is not abated by IL-6 blockades.^32^ On a fundamental level, the patient's suffering should be minimized throughout the treatment, and cytokine toxicities should be avoided as soon as possible before they endanger the patient. In this review, we discuss six distinct strategies that consistently exercise greater regulation over cytokine production, with major implications for preemptively preventing the onset of CRS in CAR-T cell therapy (Table 1).

**Table 1:** Summary of the presented strategies to curtail CRS associated with CAR-T therapy

Classification	Short description	Ref.
**Adaptor-based strategies**	α-FITC CARs: Administration of folate-FITC adaptors controls the over-activity of T cells expressing anti-FITC CARs. Cancer cells, such as lung and ovarian, that express folate receptors can be targeted by this approach.	37 and 45
	SpyCatcher–SpyTag: The anti-TAA-SpyTag fusion protein forms a covalent bond with both a chimeric fusion receptor of SpyCatcher and an intracellular T cell activation domain expressed on T cells, enabling the engineered T cells to lyse tumor cells.	46 and 47
	SUPRA CAR: Controlled activation of T cells expressing a universal T cell receptor “zipCAR”, comprised of an extracellular leucine zipper and intracellular signaling domains, is achieved by supplementation of a fusion protein, zipFv: an anti-TAA scFv and a leucine zipper.	41

**Orthogonal cytokine-cytokine-receptor pairs**	Orthogonal IL-2 and IL-2Rβ pairs: Engineered bioorthogonal IL-2/IL-2Rβ pairs enable highly specific activation of adoptively transferred T cells without activation of host cells expressing WT IL-2Rβ.	64–66

**Regulating macrophages’ cytokine activity**	Interrupting catecholamine loop: Atrial natriuretic peptide (ANP) and metyrosine (MTR) inhibit the self-amplifying feed-forward catecholamine loop in macrophages, showing potential as inhibitors of CRS without deteriorating CAR-T cell activity.	72
	Inactivation of GM-CSF: Genetic ablation of GM-CSF in CAR-T cells as well as administration of a neutralizing antibody against GM-CSF (lenzilumab) have the potential to prevent macrophage differentiation and subsequent production of pro-inflammatory cytokines.	80 and 81

**Autonomous neutralization of key cytokines**	Secretion of soluble antagonists: CAR-T cells engineered to secrete IL-1 receptor antagonist (IL-1Ra) and/or anti-IL-6 scFv autonomously block key cytokine signaling pathways involving CRS.	71, 85 and 87
	Receptor-based neutralization: The non-signaling membrane receptor of IL-6 expressed on CAR-T cells can efficiently trap systemic IL-6 without affecting CAR-T cell function.	88

**Kill switches**	Suicide genes: Introduction of a suicide gene, such as inducible caspase 9 (iCasp9) or herpes simplex virus tyrosine kinase (HSV-TK), into CAR-T cells enables the removal of CAR-T cells by the corresponding drugs.	92, 93, 95–98 and 100
	Target antigens of approved antibodies: Antigens such as EGFR and CD20 can be expressed on CAR-T cells to achieve elimination of CAR-T cells with clinically approved antibodies.	101–104

**Reversible suppression of CARs**	Inhibition of CAR Kinases: Dasatinib reversibly inactivates CAR-T cell function through inhibiting phosphorylation of tyrosine-based activation motifs (ITAMs) within TCR-based immunoreceptors such as CD3ζ and ZAP70.	108 and 114
	Ligand-induced degradation (LID): The LID domain is comprised of an FKBP12 mutant (F36V) with a short cryptic degron (19-amino-acid peptide) and can be fused to the intracellular domain of the CAR. The addition of its ligand, shield-1 or aquashield-1 (AS-1) triggers exposure of the degron, enabling proteasomal degradation of the CAR-LID fusion protein and inactivation of CAR-T cells.	109
	Proteolytic-targeting chimera (PROTAC): T cells modified to express a CAR-bromodomain (BD) fusion protein can be inactivated by exogenous supplementation of PROTAC compounds targeting the BD.	110
	Hypoxia-sensing CAR: Stringent control over expression of the CAR only under the hypoxic conditions is achieved by designing a hypoxia-responsive promoter that drives CAR expression and fusion of an oxygen-dependent degradation domain (ODD) of hypoxia-inducible factor-1 alpha (HIF1α) to the CAR.	117–120

## 2. Adaptor-based strategies

**Fig. 1:**
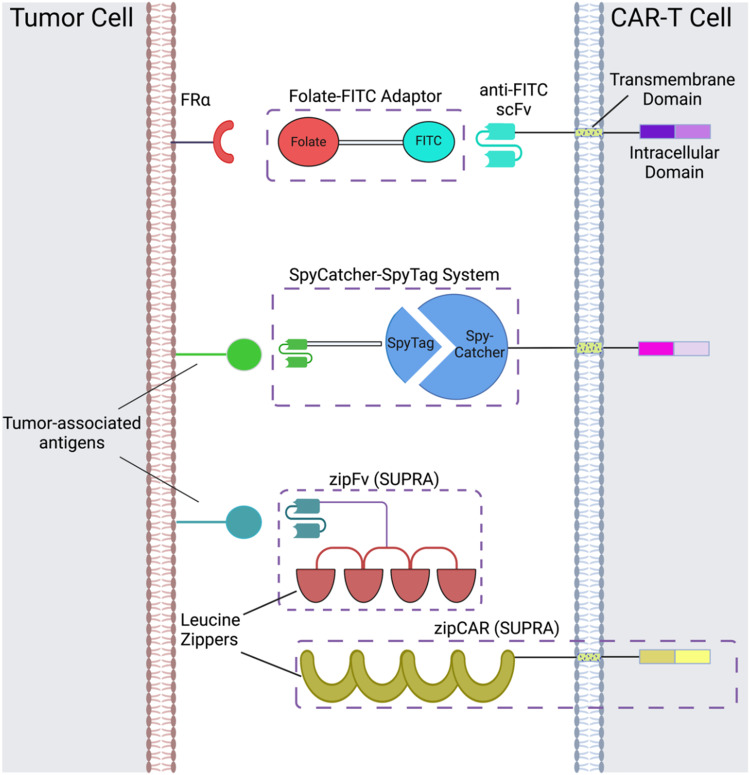
CAR adaptor-based strategies include the folate-FITC adaptor, conjugation between the SpyCatcher and SpyTag peptides, and interactions between the leucine zippers of zipFvs and zipCARs of the SUPRA CAR-T cell system. Created with https://BioRender.com.

CAR adaptor systems are composed of adaptor CARs expressed on engineered T cells and tumor-specific adaptor molecules. The adaptors link TAAs and CAR-T extracellular domains; in turn, the CAR-T cells are designed to target a binding agent on the adaptor (Fig. 1). This method is different from conventional CAR-T cell therapy, in which all the components of CAR activation directly bind to each other (*e.g.* the scFv directly binds to the TAA to induce CAR activation).

There are two important benefits of adaptor-based CAR systems: firstly, the approach represents a logical AND gate, in that both TAA and the adaptor must be present for the CAR-T cell to become activated (from a previously inactivated state) and lyse tumor cells. If systemic cytokine concentrations and inflammation become life-threatening for the patient, researchers can pause the treatment, by temporarily suspending adaptor administration or introducing competitive inhibitors. Secondly, the adaptors have the capacity to be engineered for various targets; this quality of universality allows researchers to bypass re-engineering CAR-T cells to target different TAAs, which is a time-intensive procedure; rather, the adaptor alone can be modified to target different molecules, while the CAR-T cells remain intact.

Originally, the adaptor-based strategy for control over T cell activity was reported using a CAR against the fragment crystallizable region (Fc) and anti-TAA antibody.^33^ Since then, a variety of CAR adaptor systems have been developed.^34–47^ Among these, we will highlight prominent examples of CAR adaptor systems that focused on testing the safety and controllability of CAR-T cells, to prevent progression towards CRS (Fig. 1).

### 2.1 α-FITC CARs

One subclass of CAR-adaptor systems is anti-FITC (α-FITC) CARs, in which the CAR-T cell's scFv recognizes fluorescein isothiocyanate (FITC).^45^ A specific branch of α-FITC CARs is folate-FITC adaptors: CAR-T cells target FITC on the adaptor, which also contains folate. The vitamin folate is chosen because it can bind with a high affinity to the folate receptor (FR), whose alpha variant (FRα) is expressed on nearly half of all cancers, such as lung and ovarian.^37,48^ Folate is essential for maintaining DNA production in cells, especially for tumor cells, which replicate uncontrollably.^49^ Consequently, FRα is highly specific to tumor cells and has a lower expression on normal cells, making it a promising target for tumor-localized treatments.^50,51^

**Fig. 2:**
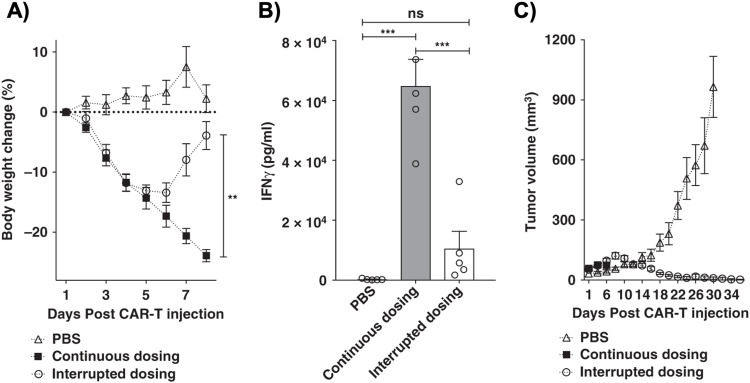
Control of CRS intensity by interruption of bispecific adapter administration. (A) Analysis of bodyweight change (%) as a measure of CRS intensity after administration of a high dose of anti-fluorescein CAR T cells (15  ×  10^6^) in either the absence (PBS) or presence of FITC-folate (500 nmole kg^−1^ administered on days 1, and 2, and alternate days thereafter). In the interrupted dosing regimen, the continuous dosing schedule was followed except FITC-folate injections were omitted on days 4 and 6. (B) Analysis of IFNγ levels in mouse plasma on day 6 using the dosing regimens described in (A). (C) Measurement of tumor volumes in mice treated as described in (A). *n*  =  5 mice per group. Data represent mean  ±  s.e.m. One-way ANOVA with *post hoc* Tukey tests were performed for all comparisons (*denotes a *p*-value  < 0.05, ** < 0.01, *** < 0.001, ns  =  not significant). From *Nature Communications*, **10**, Y. G. Lee, H. Chu, Y. Lu, C. P. Leamon, M. Srinivasarao, K. S. Putt and P. S. Low, 2681, Copyright (2019). Reprinted under a Creative Commons Attribution 4.0 International License (https://creativecommons.org/licenses/by/4.0/).

The administered dosage of folate-FITC adaptors, in tandem with α-FITC CARs, greatly affected cytokine concentrations and tumor killing in murine models.^45^ For example, interrupting adaptor administration enabled the continuation of CAR-T cell therapy and reversed pre-existing symptoms of CRS (Fig. 2). In the absence of folate-FITC adaptors, CAR-T cells did not perform tumor cell killing, thus demonstrating the additional level of security provided by the adaptors.^45^ Furthermore, introducing excess folate outcompeted the adaptor for the tumor FR, while excess fluorescein outcompeted the adaptor for the anti-FITC CAR-T cells. As a result of these competition assays, the adaptor had reduced binding to FR and CAR-T cells, with lower cytokine concentration and tumor cell lysis.^37,45^ Utilizing competitive inhibition offers a built-in approach to preemptively curtail the inception of CRS; for example, if concentrations of cytokines begin to reach dangerously high levels, competitive inhibitors can be introduced to halt CAR-T cell activity, and thus cytokine production.

Since folate-FITC CAR therapy is regulated by adaptor dosage, administration techniques should be rigorously tested before clinical applications. For example, Lee *et al.* tested various treatment modifications, such as varying the administration frequency, interruptions, progressive increments, *etc.*, to fully optimize the CAR-T cells’ efficacy *in vivo* and alleviate cytokine toxicity.^45^ The process provides insight into how the adaptor could realistically be administered in the clinic.

### 2.2 SpyCatcher–SpyTag

Another adaptor strategy capitalizes on the SpyCatcher–SpyTag system, which has been widely used for protein bioconjugation applications.^52–55^ The SpyCatcher and SpyTag peptides are derived from regions of the N and C-termini, respectively, of the collagen adhesin domain of a fibronectin-binding protein from the bacterium, *Streptococcus pyrogenes*; the high affinity between the separate SpyCatcher and SpyTag results in the formation of a peptide bond *via* Asp117 and Lys31, respectively.^52^ Immunoglobins and scFvs can be introduced onto the SpyTag molecule, which binds to the SpyCatcher receptor expressed on engineered T cells. The conjugation of the SpyCatcher and SpyTag forms an adaptor molecule that acts as a bridge between the scFv and CAR extracellular domains. This approach has been utilized by multiple groups,^46,47^ with evidence of reduced and controlled pro-inflammatory cytokine concentrations depending on scFv-SpyTag dose and comparable anti-tumor efficacy with conventional CAR-T cells *in vitro* and *in vivo*.^46^ Additionally, the carrier T cells can be designed to target multiple antigens at once, by taking advantage of the universality of the SpyTag and SpyCatcher immune receptors.^47^ A major consideration when designing CAR-based adaptors is their half-life, which determines the appropriate dosage administration and potential toxicities that occur in the treatment's duration. The SpyTag carrying the scFv has a short half-life *in vivo*. This property could be beneficial for preventing CAR-T cells from remaining activated in the body for extended periods of time, which decreases the probability of developing CRS.^47^

### 2.3 SUPRA CAR-T cells

Split, universal, and programmable (SUPRA) CAR-T cells are a paradigm of CAR controllability and tuning.^41^ Developed by Cho *et al.*, the SUPRA CAR system contains a zipFv, a composite structure consisting of an scFv and a leucine zipper, and a universal T-cell receptor (known as zipCAR) which comprises signaling domains inside the cell and an extracellular leucine zipper.^41^ Following a similar principle as the SpyCatcher–SpyTag system, when the zipFv and zipCAR bind together *via* the leucine zippers, along with the TAA, the SUPRA CAR system becomes activated. Therefore, to target multiple TAAs, one only needs to modify the zipFv (to contain the appropriate scFvs), as opposed to genetically re-engineering the whole CAR-T cell for targeting each antigen directly. Furthermore, designing zipFvs and zipCARs using mutually orthogonal leucine zipper pairs allows precise regulation of CAR-T cells activity based on multiple TAA recognition.^41^

**Fig. 3:**
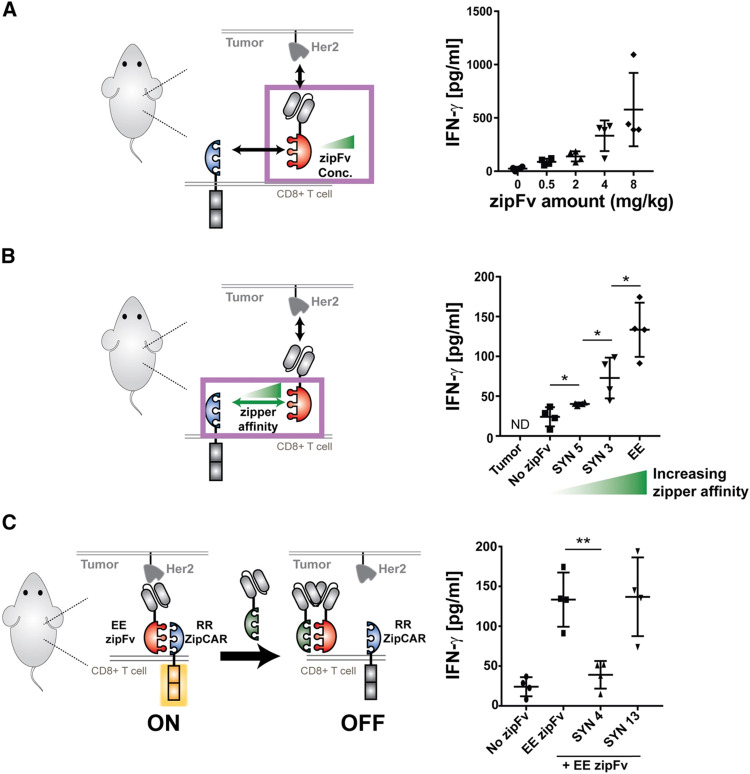
*In vivo* control of cytokine production by the SUPRA CAR. (A) *In vivo* IFN-γ cytokine level at 24 h. The i*n vivo* cytokine level increased in a dose-dependent manner (*n* = 4, mean ± SD). (B) The *in vivo* IFN-γ cytokine level at 24 h, demonstrating a leucine zipper affinity-dependent increase of *in vivo* IFN-γ cytokine (*n* = 4, mean ± SD). (C) The i*n vivo* IFN-γ cytokine level demonstrating the effect of competitive (SYN4) or non-competitive (SYN13) zipFv (the no-zipFv EE conditions are the same as the groups shown in B; *n* = 4, mean ± SD, statistical significance was determined by Student's *t*-test, * = *p* ≤ 0.05, *** = *p* ≤ 0.001). Reprinted from *Cell*, **173**, J. H. Cho, J. J. Collins and W. W. Wong, Universal Chimeric Antigen Receptors for Multiplexed and Logical Control of T Cell Responses, 1426–1438, Copyright (2018), with permission from Elsevier.

In the study, when regulating SUPRA CAR activity, several key parameters were considered: the affinities between two leucine zippers and between the scFv and TAA, zipFv concentration, and zipCAR expression; these factors are crucial for modulation of CAR activation and cytokine concentrations and have the potential to act as barriers against unbridled cytokine production (Fig. 3A and B). Furthermore, the introduction of competitive zipFvs that bind to the leucine zipper of the original zipFv prohibit zipFv–zipCAR interaction, thus successfully inhibiting SUPRA CAR-T cell activation (Fig. 3C). This competition assay has major implications for prohibiting CRS by changing the dosage of free zipFvs.

Like the SUPRA strategy, combinatorial antigen recognition has paved the way for more precise activation of CAR-T cells when interacting with tumors. Specifically, the system of multiple co-stimulatory domains (such as CD3ζ and CD28) which support multiple scFvs that target individual TAAs acts as an AND gate for CAR activation only when both TAAs are present. Indeed, the technique of “dual-targeting” has been successfully implemented in CAR-T cells against breast cancer (antigens ERB2 and MUC1)^56^ and prostate cancer (prostate-specific membrane antigen and prostate stem cell antigen).^57^ Furthermore, *de novo* protein switches have been developed to harness combinatorial antigen recognition to create logic gates that regulate CAR-T cell activity when interacting with TAAs.^58^ Incorporating logic-based therapies with masking peptides for tumor-specific activation would also be a promising approach to localize CAR-T cell function to the tumor.^59^ With increased control over CAR-T activity, there is an opportunity for synergy between SUPRA CAR-T cells and similar strategies to fine-tune logic circuits that can safely and effectively harness CAR-T cytokine production to further minimize CRS.

These adaptor-based strategies are heavily dependent on dosage and half-lives, which limits the scope for unbridled cytokine storms over long periods of time. Furthermore, unlike conventional CAR-T cells that only require the TAA for activation, additional components from the adaptors are necessary for the activation of adaptor-based CAR-T cells. Competitive inhibitors can also be introduced in a dose-dependent fashion to pause the therapy if cytokine concentrations reach dangerous levels. Finally, there is potential for developing optimal administration schedules that can preemptively curtail CRS before it even occurs in the patient.

## 3. Orthogonal cytokine–receptor pairs

Interleukin-2 (IL-2), a cytokine that promotes T cell expansion and anti-tumor activity, was FDA-approved (as “aldesleukin”) for treating renal cell carcinoma (in 1992) and melanoma (in 1998).^60^ Despite its potential, it is notorious for its toxicity, leading to capillary leak syndrome and damaging organs, such as the heart and lungs.^61^ Due to its propensity to systemically expand T cell populations, IL-2 can be concomitantly administered to support adoptively transferred T cells. IL-2 can, however, also lead to CRS, which is associated with a higher number of CAR-T cells present.^62^ Furthermore, IL-2 also promotes the proliferation of the immunosuppressive regulatory T (Treg) cells,^63^ a phenomenon which hinders the anti-tumor efficacy of other immune cells. To reduce both the toxicity of IL-2 and CRS, synthetic IL-2 receptor-beta (IL-2Rβ) and IL-2 pairs were developed to be used as an orthogonal system to increase the specificity and controllability of adoptive cell therapy.^64–66^

**Fig. 4:**
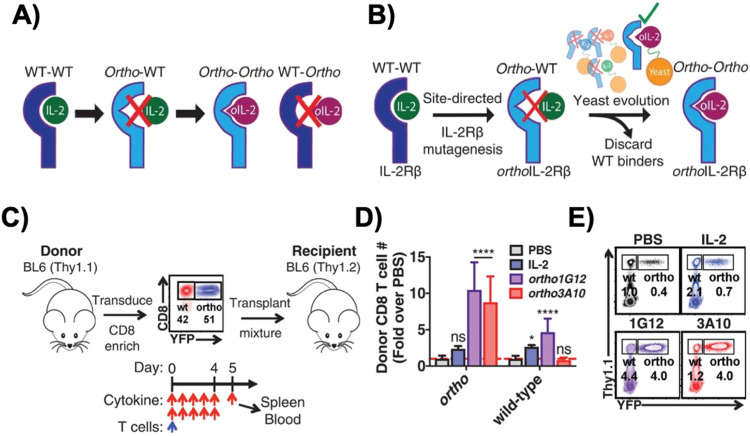
Engineering of orthogonal IL-2 and IL-2R pairs. (A) Schematic overview of orthogonal IL-2/IL-2R pairs, consisting of a mutant IL-2 cytokine and mutant IL-2R that interact specifically with each other but do not cross-react with their wild-type counterparts. (B) The strategy used to engineer orthogonal IL-2/IL-2Rβ pairs. (C) Schematic of the adoptive CD8^+^ T cell transplant mouse model. (D) Quantification of donor wild-type and *ortho* CD8^+^ T cells in the spleen of recipient mice treated twice daily with phosphate-buffered saline (PBS), IL-2 (250 000 IU per dose), *ortho*IL-2 1G12 (250 000 IU per dose), or *ortho*IL-2 3A10 (2 500 000 IU per dose). (E) Representative flow cytometry data quantified in (B) depicting donor (Thy1.1^+^) wild-type (YFP^−^) and *ortho*IL-2Rβ (YFP^+^) CD8^+^ T cells in the spleen of recipient mice. From *Science*, **359**, J. T. Sockolosky, E. Trotta, G. Parisi, L. Picton, L. L. Su, A. C. Le, A. Chhabra, S. L. Silveria, B. M. George, I. C. King, M. R. Tiffany, K. Jude, L. V. Sibener, D. Baker, J. A. Shizuru, A. Ribas, J. A. Bluestone and K. C. Garcia, Selective targeting of engineered T cells using orthogonal IL-2 cytokine–receptor complexes, 1037–1042. Copyright (2018). Reprinted with permission from AAAS.

Originally developed in 2018 by Sockolosky *et al.*, IL-2 and IL-2Rβ were mutated and evolved in multiple yeast surface display libraries, to select for murine orthogonal IL-2 (ortho-mIL-2) by evaluating its affinity to murine orthogonal IL-2Rβ (ortho-mIL-2Rβ), and not wild-type (WT) IL-2Rβ (Fig. 4A and B).^64^ CD8^+^ murine T cells were transduced with selected mutants of ortho-mIL-2Rβ. When the WT or ortho-IL-2 (variant 3A10 lacking wild-type IL-2Rβ signaling) was introduced, the latter increased phosphorylation of the transcription factor STAT5 in transduced T cells but not in wild-type T cells, thus prompting further proliferation of the transduced population *in vivo* (Fig. 4C–E). Moreover, the activity of T cells was regulated by the dose of ortho-mIL-2 for interacting with the ortho-mIL-2Rβ receptor on immune cells.

To expand on this previous work, Zhang *et al.* implemented a cytokine–cytokine receptor pair system in conjunction with human CAR-T cells, demonstrating its ability to specifically modulate CAR-T cell activity.^65^ To generate the human orthoIL-2Rβ (ortho-hIL-2Rβ), H133D and Y134F mutations were introduced to WT hIL-2Rβ to abolish its binding to hIL-2. Then, ortho-hIL-2 was obtained after multiple rounds of functional screening and extraction of key residues from ortho-mIL-2. Antigen-stimulated CAR-T cells expressing ortho-hIL-2Rβ underwent considerable cellular expansion, which was positively correlated with the experimental daily dose of ortho-hIL-2 administered to mice.

Ortho-hIL-2Rβ^+^ CAR-T cells were administered to xenograft CD19^+^ Nalm6 leukemia murine models, followed by administration of ortho-hIL-2 daily or every other day for 2 weeks.^65^ Higher doses of ortho-hIL-2 led to weight loss and higher mortality rates, and experiments with NSG mice indicated that lower doses led to more effective leukemia control with fewer instances of mouse death. Even with lower CAR-T cell doses, ortho-hIL-2 was shown to radically advance the anti-leukemic response, but with increased mortality rates among mice.^65^ The observed toxicity was accompanied by infiltration of activated CAR-T cells into healthy tissues, and it is suggested that both CAR or TCR-mediated T cell activation and ortho-hIL-2 administration are required for the observed toxicity. However, the detailed mechanism underlying the toxicity of Ortho-hIL-2Rβ^+^ CAR-T cells remains elusive. Lastly, after leukemia progression during which CAR-T cells could only temporarily and insubstantially hinder the cancer, introducing the ortho-hIL-2 in conjunction with Ortho-hIL-2Rβ^+^ CAR-T cells drastically augmented the immunotherapy, with all mice treated in this fashion achieving a complete response with higher CAR-T cell numbers.^65^

**Fig. 5:**
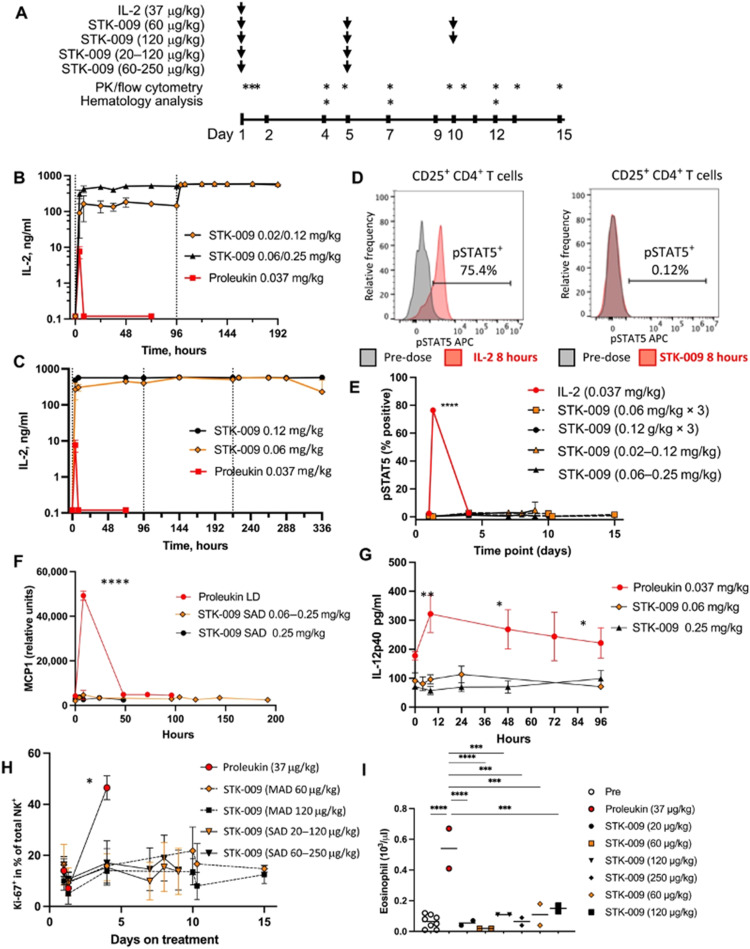
STK-009 does not induce IL-2-mediated toxicity in nonhuman primates. (A) Treatment and analysis schedule for WT hIL-2 and STK-009 dosing cynomolgus monkeys. Subcutaneous treatment with hIL-2 and STK-009 is indicated by the arrows. Blood draws for pharmacokinetic (PK) analysis, cytokine analysis, and flow cytometry are indicated by asterisks. *N* = 2 cynomolgus monkeys per treatment group. (B) The serum concentration of IL-2 (aldesleukin) and STK-009 was measured after subcutaneous injection of WT hIL-2 on day 1 or STK-009 on days 1 and 5. (C) Serum concentration of hIL-2 (aldesleukin) and STK-009 was measured after subcutaneous injection (STK-009 three doses, days 1, 5, and 9). (D and E) Flow cytometry analysis of CD25^+^CD4^+^ T cells for phosphorylated STAT5 (pSTAT5). (D) Representative histogram for pSTAT5 8 hours after treatment with hIL-2 (left) and STK-009 (right). I Time course of pSTAT5^+^ CD25^+^ CD4^+^ T cells over the 2 week study. (F and G) Serum MCP-1 (F) and IL-12p40 (G) concentrations for the first 4 days after IL-2 or STK-009 treatment. (H) NK cell proliferation (Ki-67^+^ NK cells) was measured in the blood for 2 weeks after hIL-2 or STK-009 treatment. MAD, Multiple Ascending Dose; SAD, Single Ascending Dose. (I) Eosinophil count in the blood was quantified on day 4 after hIL-2 or STK-009 treatment. Data are presented as means ± SEM. These studies have been repeated yielding similar results. Kruskal–Wallis tests with Tukey's correction for multiple comparisons were performed I(E) to (I) and a one-way ANOVA with Dunn's correction for multiple comparisons was performed in (H). *P < 0.05, **P < 0.01, ***P < 0.001, and ***P < 0.0001. From *Science Translational Medicine*, **13**, P.-J. Aspuria, S. Vivona, M. Bauer, M. Semana, N. Ratti, S. McCauley, R. Riener, R. d. W. Malefyt, D. Rokkam, J. Emmerich, R. A. Kastelein, P. J. Lupardus and M. Oft, An orthogonal IL-2 and IL-2Rβ system drives persistence and activation of CAR T cells and clearance of bulky lymphoma, eabg7565, Copyright (2021). Reprinted with permission from AAAS.

Scientists at Synthekine, a cytokine engineering company founded by Professor K. Christopher Garcia, conducted a study implementing his research on ortho-IL-2/IL-2Rβ in non-human primates and murine advanced lymphoma models.^66^ In the study, subcutaneous repeat dosing of the polyethylene glycol-modified (pegylated) ortho-hIL-2 (denoted as STK-009) in cynomolgus monkeys (Fig. 5A), which possess highly conserved ligand binding residues in IL-2Rβ compared to a human, demonstrated a prolonged *in vivo* half-life of STK-009 (Fig. 5B and C). The authors mentioned that this is presumably due to the lack of STK-009's binding to WT hIL-2Rβ in addition to the effect of pegylation. STK-009 did not induce IL-2-mediated biological responses (Fig. 5D–G). STK-009 did not activate cellular populations related to IL-2 mediated toxicity such as NK cells and eosinophils (Fig. 5H and I). Anti-CD19-CD28ζ CAR-T cells expressed ortho-hIL-2Rβ and targeted Raji B-cell lymphoma in SCID mice; STK-009 was introduced to expand only the Ortho-hIL-2Rβ^+^ CAR-T cells. The orthogonal system achieved complete responses in mice with large lymphomas, with 100 times the expansion of ortho-hIL-2Rβ^+^ CAR-T cells with STK-009 compared with the control.^66^ After STK-009 administration was ended upon tumor eradication, CAR-T cell numbers decreased at a rate dependent on their immune cell phenotypes.

Aspuria *et al.* emphasize the STK-009 therapy's future potential to minimize the severity of cytokine toxicity.^66^ The combination of STK-009 and Ortho-hIL-2Rβ^+^ CAR-T cells induced upregulation of CRS biomarkers like IL-6 and temporary weight loss (regained after suspension of STK-009 administration) in immunodeficient mice; these CRS-like symptoms were attributed to antigen-specific activation and expansion of CAR-T cells.^66^ Since CRS is a byproduct of elevated systemic cytokine concentrations and cellular expansion, the authors suggest that taking advantage of STK-009's ability to instigate potent tumor clearance with lower CAR-T cell numbers would decrease the risk of CRS and retain therapeutic effectiveness.^66^

## 4. Regulating macrophages’ cytokine activity

Macrophages are immune cells that possess a variety of functions, including phagocytizing foreign substances and producing pro-inflammatory cytokines. CAR-T cells interact with macrophages: Although interactions between the CD40 ligand on T cells and CD40 receptor on macrophages have been studied, further investigation into the precise mechanisms of T cell-induced activation of macrophages is warranted.^67^ Antigen-stimulated CAR-T cells activate macrophages, which produce and react with catecholamines *via* alpha adreno-receptors to produce pro-inflammatory cytokines, such as IL-6, IFN-γ, IL-1, *etc.*, leading to systemic inflammation, fever, and organ damage; this phenomenon has been widely regarded as the core of CRS in CAR-T cell therapy.^68,69^ In fact, monocytes and their derived macrophages are significantly more powerful sources of pro-inflammatory cytokines, compared to CAR-T cells themselves.^69–71^ Here, we discussed approaches that regulate macrophages’ cytokine activity to curtail CRS.

### 4.1 Interrupting catecholamine loop

Staedtke *et al.* constructed a model for the macrophages’ “catecholamine self-amplifying feed-forward loop,” an integral element of CRS.^72^ Tyrosine hydroxylase (TH) is an enzyme of catecholamine biosynthesis.^73,74^ Activating the TH gene stimulates cytokine release from macrophages (and probably T cells) and production of catecholamine (adrenaline, noradrenaline and dopamine). Catecholamine stimulates the cell's adrenergic receptors, which activates the TH gene again. As such, TH is a major component in macrophages’ feed-forward loop.^72^

**Fig. 6:**
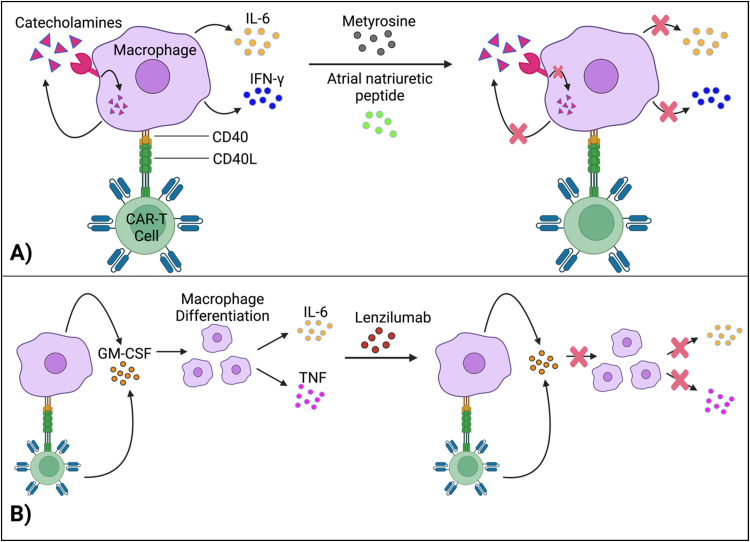
Methods for regulating macrophages’ cytokine activity. (A) Interruption of the feed-forward catecholamine cycle with metyrosine and atrial natriuretic peptide, thus reducing cytokine concentrations. (B) Lenzilumab neutralizes GM-CSF, which further inhibits pro-inflammatory cytokine production. Created with https://BioRender.com.

Atrial natriuretic peptide (ANP) and metyrosine (MTR) act as antagonists against the catecholamine loop (Fig. 6A). ANP is a hormone released by the heart's atria to lower blood pressure, while MTR is a drug that combats hypertension and high blood pressure. In murine models, both ANP and MTR inhibited macrophages’ catecholamines.^72^ While ANP's mechanism is undetermined, MTR acts by directly blocking TH, an enzyme required for catecholamine synthesis. CD19^+^ human Burkitt's lymphoma-bearing immunodeficient mice (retaining macrophages) treated with both MTR and anti-CD19 CAR-T cells (hCART19) had augmented survival rates and significantly lower levels of catecholamines and inflammatory cytokines, compared to mice treated with CAR-T cells only.^72^ Similarly, the combined use of either ANP or MTR with CAR-T cells reduced the systemic release of catecholamines and inflammatory cytokines while retaining the therapeutic efficacy of CAR-T cells in a syngeneic mouse leukemia model.

Although ANP and MTR offer promising solutions for uncoupling macrophage-induced CRS from CAR-T cell efficacy, more investigation is needed to delve into the intricacies of this loop. For example, the mechanisms of how ANP decreases catecholamine production, without significantly impairing CAR-T cell activity, need to be investigated to give direct insight into uncoupling CAR-T cell toxicity from function. Comprehending these molecular pathways is essential for clinical translation, especially for understanding potential physiological deviations between mouse models and human cells.

### 4.2 Inactivation of GM-CSF

Granulocyte-macrophage colony-stimulating factor (GM-CSF), a glycoprotein, acts as both an inflammatory cytokine and growth factor for myeloid cells.^75,76^ GM-CSF activates macrophages by increasing their sensitivity to macrophage colony-stimulating factors.^77^ Leukocytes, such as macrophages and T cells, produce GM-CSF.^75^ Additionally, IL-1, IL-12, and tumor necrosis factor (TNF) are pro-inflammatory cytokines that stimulate GM-CSF expression.^78^ In turn, by promoting macrophage differentiation (among other methods), GM-CSF leads to the production of IL-6, TNF, IL-1-beta, *etc.*,^69,79^ which are significant, pro-inflammatory biomarkers for CRS.

Gene editing techniques such as TALEN and CRISPR-Cas9 have been utilized to engineer CAR-T cells with a GM-CSF genetic knockout with reduced production of pro-inflammatory cytokines (IL-6) and chemokines (chemokine ligand 2).^80,81^ Moreover, lenzilumab, a GM-CSF-neutralizing monoclonal antibody, achieved similar results and even augmented CAR-T cell proliferation *in vitro* (Fig. 6B).^81^ More investigation is needed to understand how inactivating GM-CSF, a growth factor, enhances cellular proliferation. Additionally, lenzilumab is currently being tested alongside CAR-T cells in Phase 1 and Phase 2 clinical trials to evaluate the combination treatment's toxicity and efficacy against B-cell lymphoma (NCT04314843).

Even though GM-CSF has been implicated in various auto-immune diseases, such as multiple sclerosis,^82^ it has also been tested in murine models to treat other auto-immune diseases, such as Type I diabetes,^83^ and myasthenia gravis,^84^*etc.* It is also being tested in clinical trials for efficacy against hematological illnesses, such as peripheral arterial disease (NCT03304821). Although GM-CSF inhibition has demonstrated lower cytokine toxicity for CAR-T cells, in clinical applications, it is essential to consider how GM-CSF-inhibited CAR-T cells will affect particularly immune-compromised patients with other pre-existing conditions.

Since macrophage-induced cytokine production has been regarded as the cornerstone of CRS, placing safeguards on macrophages can make CAR-T cell therapy safer for the patient. However, it is crucial that controlling the cytokine production does not impede the anti-tumor efficacy of the CAR-T cells. Accounting for adverse side events is especially important for GM-CSF inhibition, which could be detrimental for patients with other immunological diseases. Thus, even though macrophages should remain immunotherapeutic targets for CRS, more investigation is needed to successfully validate safe approaches that decrease their cytokine levels.

## 5. Autonomous neutralization of key cytokines

As CRS is initiated when CAR-T cells are most rapidly expanding in patients, it would be beneficial to synchronize the supply of CRS therapeutics with CAR-T cell proliferation. Here, we highlight several examples of engineered CAR-T cells designed to achieve this feat without additional intervention during the therapy.

### 5.1. Secretion of soluble antagonists

Giavridis *et al.* genetically engineered T cells to simultaneously express anti-CD19 CAR and IL-1 receptor antagonist (IL-1Ra), anakinra.^71^ IL-1 is known as one of the crucial cytokines in worsening CRS,^70,71^ and systemic administration of anakinra successfully abrogated CRS-related mortality in a mouse model of CRS in their study. Using the same model, the authors demonstrated that the CAR-T cells armored with IL-1Ra protect mice from CRS-associated mortality. Importantly, co-expression of IL-1Ra did not hinder the CAR-T cells’ ability to secrete cytokines (*e.g.* IFN-γ, IL-2 and IL-3) or their therapeutic efficacy *in vivo*. Thus, the authors experimentally validated a new CAR-T cell design that autonomously ameliorates CRS.

**Fig. 7:**
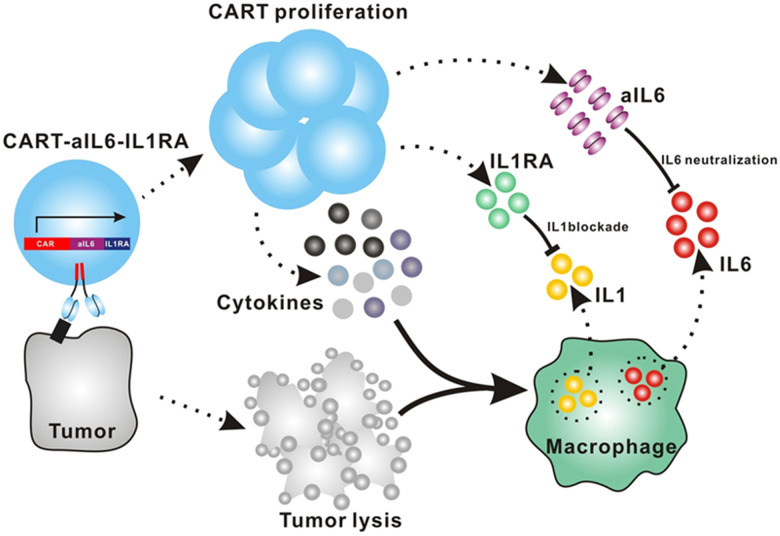
The schematic illustration of CART-secreted anti-IL-6 scFv and IL-1Ra in blocking IL-6 and IL-1 signaling during CRS of CART therapy. From *Cell Discovery*, **7**, L. Xue, Y. Yi, Q. Xu, L. Wang, X. Yang, Y. Zhang, X. Hua, X. Chai, J. Yang, Y. Chen, G. Tao, B. Hu and X. Wang, 84., Copyright (2021). Reprinted under a Creative Commons Attribution 4.0 International License (https://creativecommons.org/licenses/by/4.0/).

Similarly, Xue *et al.* recently reported the results of two clinical trials (ChiCTR2000031868 and ChiCTR2000032124) testing anti-CD19 and anti-B cell maturation antigen (BCMA) CAR-T cells which constitutively co-express anti-IL-6 scFv (derived from Sirukumab) and IL-1Ra (Fig. 7).^85^ Correlation of blood IL-1Ra concentrations with armored CAR-T expansion was observed in treated patients. IFN-γ and IL-6 concentrations are correlated with each other in conventional CAR-T therapy, reflecting the levels of CAR-T expansion and tumor cell killing.^86^ In 16 out of the 18 patients treated with CAR-T cells co-expressing anti-IL-6 scFv and IL-1Ra, the peak concentration of IL-6 was kept at a low level (<100 pg mL^−1^) whereas the peak IFN-γ concentration had varied widely (ranging from 2.6 to 4118 pg mL^−1^). The concentration of IL-1β during the treatment in the patients was also kept at a low level (<100 pg mL^−1^). 14 out of 18 patients exhibited mild (grade 1) or moderate (grade 2) CRS based on ASTCT criteria.^18^ Therefore, these CAR-T cells rendered tocilizumab dispensable during the treatment period. Finally, 90% (9 out of 10) of patients with ALL, 40% (2 out of 5) of patients with lymphoma, and 100% (2 out of 2) of patients with MM achieved a complete response, demonstrating that the therapeutic efficacy of this armored CAR-T cell design is not significantly compromised.

The burden posed by CRS is not only physical: ensuring rigorous patient monitoring during CAR-T treatment and therapeutic interventions for CRS require significant human and financial costs. To this end, the armored CAR-T design presented by Giavridis *et al.* and Xue *et al.*, which does not necessitate exogenous intervention for neutralizing key cytokines, may have the potential to deliver CAR-T cell therapy to a broader population of patients by lowering the barrier to the treatment. Xue *et al.* still observed grade 3 CRS in 4 out of 18 patients treated with the anti-IL-6/IL-1Ra expressing CAR-T cells, noting the necessity of further investigation into the involvement of other factors in CRS. Yi *et al.* recently reported a clinical trial of CAR-T cells that tested the above modifications plus KO of GM-CSF.^87^ Although the trial was small in size, this combination might be useful in further reducing the risk of CRS, as there was no grade 3 or 4 CRS in any of the 3 enrolled patients.

### 5.2. Receptor-based neutralization

**Fig. 8:**
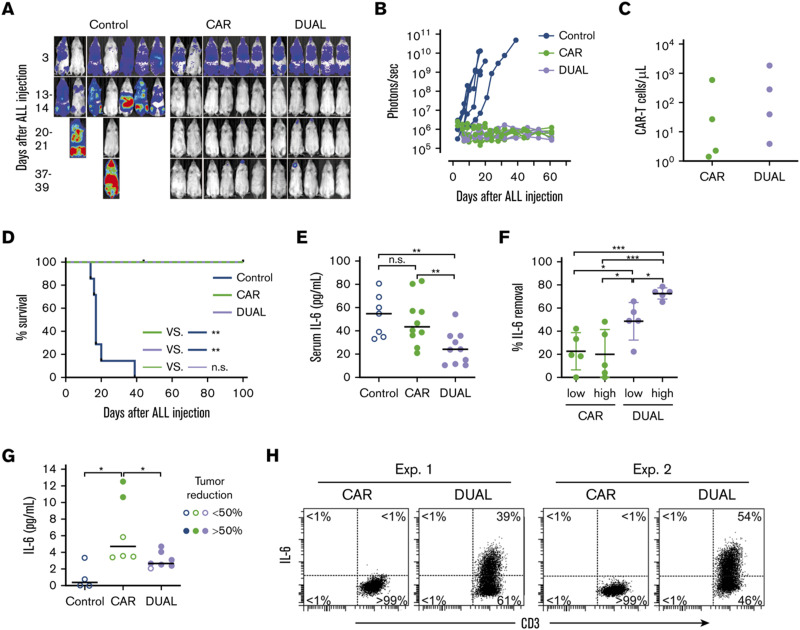
CAR-T cells expressing mbaIL-6 quench IL-6 and exert antileukemia activity in xenograft models. (A) NOD/scid-IL-2RGnull mice were injected IV with 0.5 to 1 × 10^6^ Nalm-6-luciferase cells. On day 3, mice were given T cells expressing either anti-CD19 CAR alone (85% CAR expression) or mbaIL-6 plus CAR (“DUAL”; 79% CAR expression) (20 × 10^6^ per mouse IV); all mice received 20 000 IU IL-2 IP every 2 days. Ventral images from the Xenogen IVIS-200 system after D-luciferin injection are shown. (B) Luminescence measurements (photons per second) in the mice. Each point corresponds to a measurement in 1 mouse. (C) Levels of GFP^+^ CD3^+^ CAR-T cells in blood 50 days after CAR-T cell injection in a subset of the mice. (D) Kaplan–Meier curves of overall survival for the mice shown in panel A, euthanized when the total bioluminescence signal reached 1 × 10^10^ photons per second. **P < 0.01 by log-rank test. (E) T cells expressing either anti-CD19 CAR or anti-CD19 CAR plus mbaIL-6 were injected IV in NOD/scid-IL-2RGnull mice (2–10 × 10^6^ per mouse); 3 days later, 50 ng of human IL-6 was injected IP. After 2 hours, the mice were euthanized, and serum was collected by cardiac puncture to measure the levels of human IL-6 by using ELISA. Each symbol corresponds to data from 1 mouse; bars show the mean (±SD). **P < 0.01. (F) Mice from the experiments shown in panel E were divided according to the number of T cells that were administered: 2 to 4 × 10^6^ (“low”) and 5 to 10 × 10^6^ (“high”). Values correspond to the percentage of IL-6 that was removed from the serum in each mouse, using as a reference the mean value of IL-6 measured in mice that received IL-6 with no prior injection of T cells. *P = 0.035 for CAR low *vs.* DUAL low, *P* = 0.045 from CAR high *vs.* DUAL low, *P* = 0.013 for DUAL low *vs.* DUAL high; ***P < 0.001. (G) Daudi-luciferase cells were injected IP in NOD/scid-IL-2RGnull mice (20 × 10^6^ per mouse), followed 3 days later by THP-1 and/or T cells IP (20 × 10^6^ for both cell types). Tumor engraftment was measured by *in vivo* imaging. Mice were euthanized 48 hours after THP-1 and/or T-cell injection. Symbols show IL-6 levels measured by using ELISA in peritoneal lavage, according to the percentage of tumor reduction. *P = 0.032 for CAR *vs.* no T cells; *P* = 0.046 for CAR *vs.* DUAL. (H) IL-6 binding to T cells from the peritoneal lavage of 4 mice, 2 injected with CAR-T cells and 2 injected with T cells expressing both CAR and mbaIL-6. Cells were stained with anti-mouse CD45-PE-Cy7, anti-human CD45-PerCP, anti-human CD3-APC, and anti-human IL-6-PE; the plots show selectively gated mouse CD45^−^, human CD45^+^, and human CD3^+^ cells. Modified and reprinted from *Blood Advances*, **4**, A. H. J. Tan, N. Vinanica and D. Campana, Chimeric antigen receptor–T cells with cytokine neutralizing capacity, 1419–1431. Copyright (2020), with permission from Elsevier.

Tan *et al.* recently reported a new approach to neutralize IL-6; a non-signaling membrane-bound IL-6 receptor (mbaIL-6) composed of anti-IL-6 scFv and the hinge and transmembrane domain of CD8α.^88^ Co-expression of mbaIL-6 did not change the anti-tumor efficacy (Fig. 8A–D), *in vivo* expansion, IFN-γ production and the proportion of phenotypes (naïve, effector, central memory and effector memory) of human primary CAR-T cells. mbaIL-6-expressing CAR-T cells successfully neutralized exogenously provided human IL-6 as well as IL-6 secreted by the adoptively transferred THP-1 human monocytic cell line *in vivo* (Fig. 8E–G). The capacity to neutralize IL-6 was proportional to CAR-T cell numbers. IL-6 bound to CAR-T cells was also detected by flow cytometric analysis of cells harvested from peritoneal lavage of mice treated with CAR-T and THP-1 cells (Fig. 8H). Antigen-induced activation and exhaustion profiles of the CAR-T cells [expressions of PD-1, T-cell immunoglobulin and mucin-domain containing-3 (TIM-3) and lymphocyte-activation gene 3 (LAG-3)] were not affected by mbaIL-6 expression. Further research is awaited to determine whether this strategy can prevent or ameliorate CRS.

## 6. Kill switches

Genetic and biomarker-based constructs have been developed and incorporated into CAR-T cells as a means of preemptively terminating toxicity or overactivity. We highlight key examples of suicide (or safety) switches that demonstrate specific control over CAR-T cell therapies.

### 6.1 Suicide Genes

Suicide gene systems involve transducing a T cell with a “suicide” gene, such as inducible caspase 9 (iCasp9) or herpes simplex virus tyrosine kinase (HSV-TK), and introducing an external protein to generate apoptosis of the transduced T cell. One of the most commonly tested suicide genes is the HSV-TK gene, which produces the viral enzyme, HSV-TK. When introduced with this genetic construct, the drug ganciclovir is converted to bear a triphosphate, leading to DNA chain termination and death of the cell expressing the suicide gene.^89^ Lymphocyte and hematopoietic stem cell transduction with HSV-TK and ganciclovir administration has been demonstrated to reduce toxicities, such as graft *vs.* host disease (GVHD),^90,91^ and eliminate CAR and HSV-TK-transduced cell populations in a ganciclovir dose-dependent fashion,^92^ highlighting the potential of the HSV-TK suicide gene to reduce CRS. However, HSV-TK is highly immunogenic,^93,94^ with immune responses against it decreasing transduced T cells’ persistence *in vivo*.^95^

Another strategy is the iCasp9 system, which utilizes a fusion protein consisting of the engineered enzyme, caspase-9, and the FK506 binding protein (with a mutation at the F36V residue);^96^ this fusion protein will dimerize upon the introduction of dimerization inducers,^97^ such as AP1903^93,97,98^ or AP20187,^96^ prompting apoptosis of the expressing cell *via* the caspase 3 apoptotic pathway.^99^ This approach has been commonly reported to induce apoptosis in over 90% of transduced T cell populations.^93,96,98^ Moreover, the iCasp9 genetic construct does not alter or hinder cellular anti-tumor properties, such as cytokine production.^98^ Importantly, the administration of agents such as AP1903 in conjunction with the iCasp9 construct has demonstrated efficacy in T and CAR-T cells against CRS-like symptoms, leading to reduction of pro-inflammatory cytokine concentrations (*e.g.* IL-6),^97^ recovery from body weight loss,^97^ as well as preventing the onset of GVHD.^100^

### 6.2 Target antigens of approved antibodies

An alternative approach to suicide gene switches is designing T and CAR-T cells to bear a surface biomarker or antigen that, upon the introduction of an antibody, eradicates the engineered cell. Several groups have incorporated the use of FDA-approved monoclonal antibodies to promote the lysis of cells bearing antibody targets. For example, the drug cetuximab, which targets the epidermal growth factor receptor (EGFR), has been tested against CAR-T and T cells that express cell-surface truncated EGFR (tEGFR) peptides; administered cetuximab reduced transduced cellular populations by over 95%, within as little as 24 hours in murine models.^101,102^ Other tested antibodies used for depleting CAR-T cell populations include alemtuzumab (which targets the CD52 antigen) and rituximab (which targets CD20).^103,104^ Again, administration of each antibody demonstrated rapid *in vivo* ablation of CAR-T cells bearing the relevant antigen. Capitalizing on clinically approved therapies like monoclonal antibodies can offer a safety mechanism for terminating the therapy in the onset of CRS-like symptoms. However, an important consideration is to ensure that the expressed biomarker on CAR-T cells does not affect other healthy cells or lymphocytes. For example, as the CD20 antigen is naturally present on B cells, the introduction of rituximab would lyse B cells as well as transduced CAR-T cells if they use the full-length CD20 receptor or a construct comprising the target epitope of the CD20-specific antibody,^101^ which could undermine the body's immune response to the therapy.

## 7. Reversible suppression of CARs

**Fig. 9:**
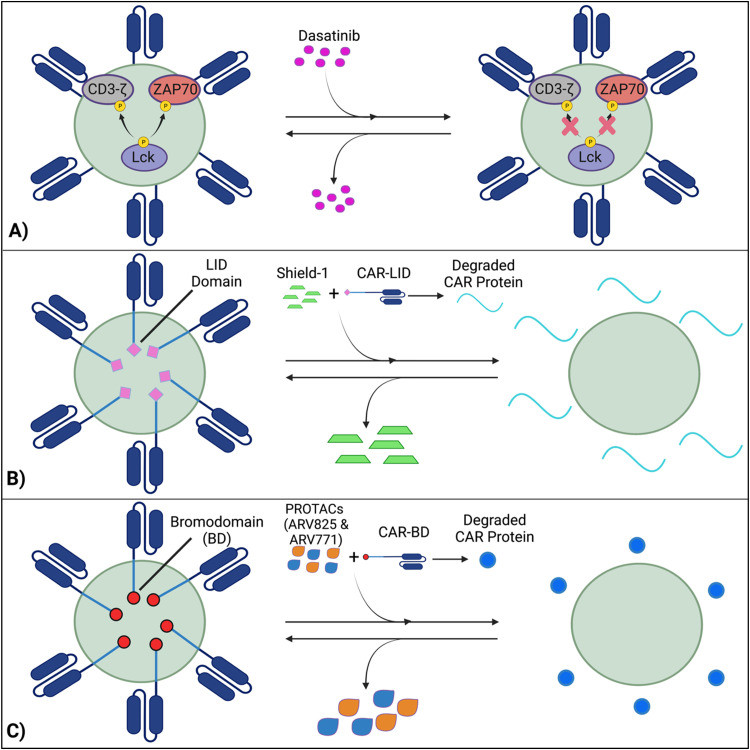
Transient suppression of CARs offers an effective alternative to completely terminating CAR-T activity, which prompts expensive and time-consuming reinfusions. (A) Dasatinib inhibits Lck-induced phosphorylation of intracellular domains, averting CAR activation, while the removal of dasatinib reverses this process. (B) A LID domain fused with the CAR protein will reversibly degrade and be restored in the presence or absence of the Shield-1 molecule. (C) With CARs fused with BDs, CAR expression will be reduced by PROTAC compounds and re-established with PROTAC removal. Created with https://BioRender.com.

Techniques like suicide genes designed to promote apoptosis in engineered cells,^96,98,100^ and antibody-mediated depletion of T cells engineered to express cell-surface antigens^102,105–107^ have been utilized as kill switches in T cell therapy to irreversibly inhibit cellular activity with the intent of preemptively stopping toxicities. However, irreversible removal of the CAR-T cells in the patient by kill switches may force re-infusions, which are expensive and time-intensive to manufacture. To facilitate smooth clinical translation, novel approaches to transiently suppress or degrade the CAR protein have been developed, which hold the potential for bypassing re-infusion procedures. Such methods involve introducing an external agent that impairs CAR-T function, while a removal restores CAR-T cell activity (Fig. 9).^108–110^ Reversible inhibition allows for greater temporal regulation to avert a systemic cytokine storm.

### 7.1 Inhibition of CAR kinases

**Fig. 10:**
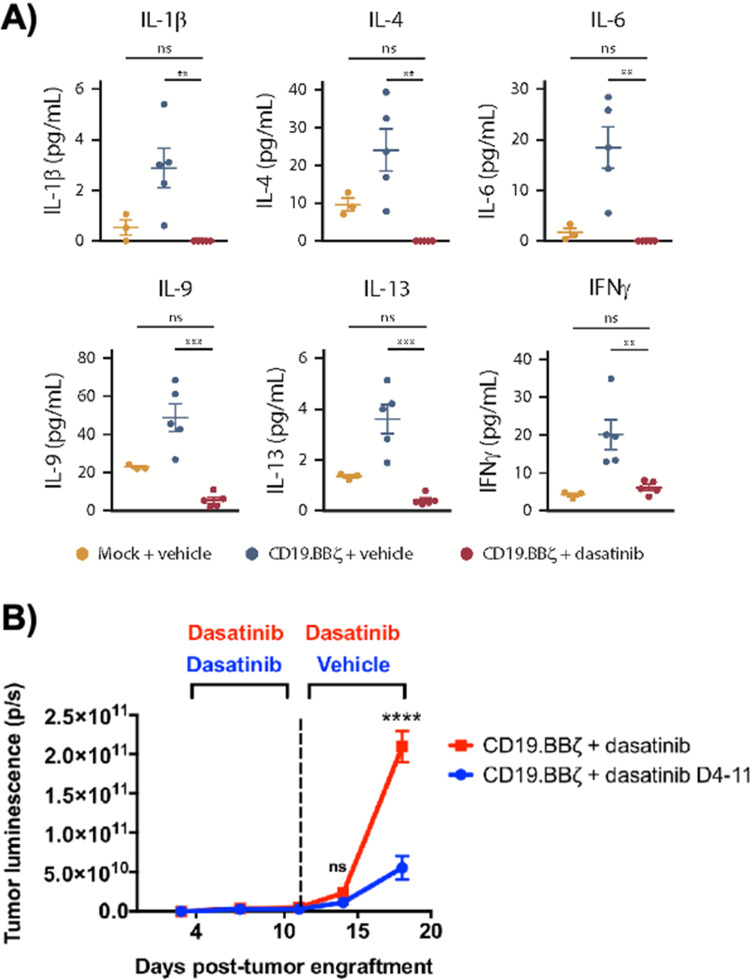
Dasatinib suppresses CAR-T cell cytokine secretion and tumor control *in vivo*. (A) 1 × 10^6^ CD19^+^ Nalm6-GL, which stably express GFP and luciferase, were engrafted into 6 to 8 week-old NSG mice *via* IV injection (*n* = 5 mice per group). At 4 days post engraftment, 1 × 10^6^ mock (untransduced) or CD19.BBζ CAR-T cells were infused *via* IV injection. Mice were subsequently dosed with 50 mg kg^−1^ dasatinib or vehicle on the day of infusion and everyday thereafter twice daily. Blood samples were collected retro-orbitally on day 3 after CAR-T infusion (day 7 post engraftment), and plasma was isolated after a brief centrifugation. Circulating concentrations of cytokines, chemokines, and growth factors were measured *via* Luminex (mock *n* = 3 mice, vehicle and dasatinib *n* = 5 mice from *n* = 1 experiment). (B) 1 × 10^6^ CD19^+^ Nalm6-GL, which stably express GFP and luciferase, were engrafted into 6–8 week-old NSG mice *via* IV injection (*n* = 5 mice per group from *n* = 1 experiment). At 4 days post-engraftment CD19.BBζ CAR-T cells were infused *via* IV injection. Mice were subsequently dosed BID with 50 mg kg^−1^ dasatinib on the day of infusion and every day thereafter (CD19.BBζ + dasatinib, red), or until 7 days post-CAR-T infusion (day 11 post-engraftment), after which mice were switched to vehicle dosing as indicated (CD19.BBζ + dasatinib D4-11, blue). Tumor growth was monitored and quantified *via* bioluminescence imaging. Modified and reprinted from *Blood Advances*, **3**, E. W. Weber, R. C. Lynn, E. Sotillo, J. Lattin, P. Xu and C. L. Mackall, Pharmacologic control of CAR-T cell function using dasatinib, 711–717, Copyright (2019), with permission from Elsevier.

Dasatinib, an FDA-approved tyrosine kinase inhibitor, has been investigated as a mechanism for reversibly inhibiting CAR-T cells to avoid CRS (Fig. 7A). Dasatinib precludes the lymphocyte-specific protein tyrosine kinase from phosphorylating immunoreceptor tyrosine-based activation motifs (ITAMs) in proteins involving T cell activation,^111,112^ such as CD3-ζ and zeta-chain-associated protein kinase 70 (ZAP70).^113^ This phenomenon temporarily inactivates CAR-T cell function including cytokine production (Fig. 10A).^108^ Moreover, upon suspension of dasatinib administration *in vivo*, CAR-T cells effectively recover their functionality (Fig. 10B),^108^ suggesting that dasatinib does not permanently impede their capability.^108,114^ Currently, an early Phase I clinical trial will test CD19 CAR-T cells and dasatinib against MM, ALL, and non-Hodgkin's lymphoma; dasatinib will be evaluated as a preconditioning agent for CAR-T cells and as a potential treatment against CRS and neurotoxicity (NCT04603872).

### 7.2 Ligand-induced degradation

Richman *et al.* designed anti-GD2 CAR-T cells co-expressing a ligand-induced degradation (LID) domain (with the LID originally constructed by Bonger *et al.*^115^) for reversible CAR expression (Fig. 7B).^109^ The LID domain consists of a human protein, FK506 and rapamycin-binding protein (FKBP) containing an F36V mutation, with an engineered cryptic degron peptide. When a ligand, Shield-1 (or the water-soluble aqua-shield (AS-1)), binds to the mutant FKBP, the degron is dislocated from its original binding position, triggering swift degradation of both the LID domain and the corresponding fused protein.^115^ Upon introduction of Shield-1 to a CAR-LID fusion protein, CAR surface expression as well as IFN-γ production significantly decreased. But a medium washout of Shield-1 almost completely restored CAR expression to its baseline. The reduction in CAR expression was dependent on the administered dosage of Shield-1 or AS-1.^109^ Lastly, the ligand-mediated control over the expansion of CAR-T cells upon antigen exposure *in vitro* and anti-tumor efficacy *in vivo* has been demonstrated.

### 7.3 PROTAC Compounds

Lee *et al.* developed a system involving proteolytic-targeting chimera (PROTAC) compounds to reversibly degrade CAR-T cells, by targeting the CAR protein, instead of the gene (Fig. 7C).^110^ After fusing bromodomains (BD) to the CAR protein, PROTAC compounds, such as ARV825 and ARV771, were introduced to degrade the BD tag, which also eliminated CAR expression on the cell surface. As such, the anti-tumor activity decreased with increasing PROTAC concentrations. Using a medium washout to remove the PROTAC compounds provided a reversible mechanism that restored the previously degraded CAR proteins.^110^

These procedures of reversible inhibition operate based on similar principles, which enable greater control and modulation over CAR-T cell activity and cytokine production and remove the constraints of patient re-infusion. Nevertheless, since the methods are heavily dose dependent, various concentrations and administration techniques should be tested to maximize functionality and minimize toxicity. For example, PROTACs of concentration 100–300 nM cause toxicity to CAR-T cells, with a 20–30% decrease in viability.^110^ Further investigation into sophisticated CAR-T regulation is warranted to curtail the risk of harming the cell population and optimize the therapies’ efficacy. Although the concept of reversible suppression itself is a promising avenue for preventing the occurrence of CRS, as CAR-T cell research moves towards solid tumors, and considering the infiltration, motion, and side effects of external agents within the tumors is critical for ensuring regulatory efficacy over CAR-T cells.

### 7.4 Hypoxia-sensing CARs

**Fig. 11:**
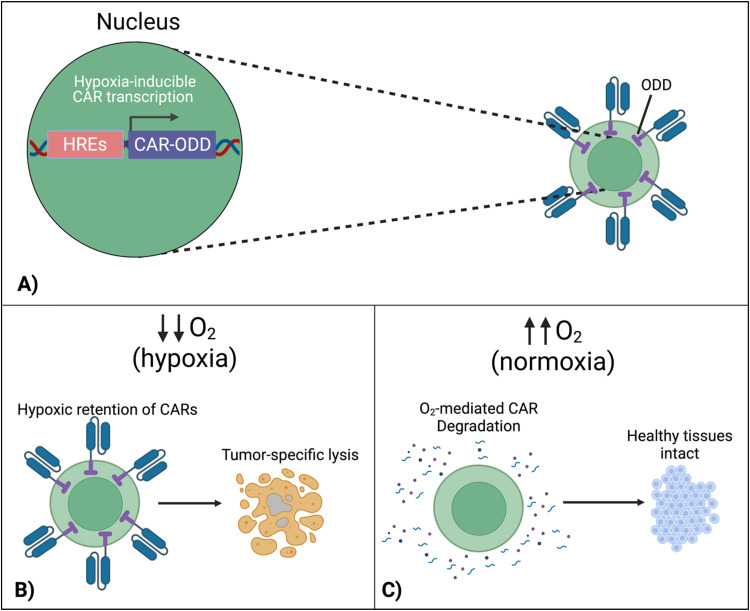
Hypoxia-sensing system for control over CAR expression. (A) Hypoxia-dependent CAR expression is achieved by combined use of hypoxia-responsive elements (HRE) and HIF1α-derived ODD. (B) CAR expression is retained in the hypoxic TME, enabling tumor cell lysis. (C) In healthy normoxic tissues, adoptively transferred T cells do not display the CAR on their surface due to its proteasomal degradation, preventing on-target, off-tumor activation and cytokine release syndrome. Created with https://BioRender.com.

To localize the effect of CAR-T cells exclusively within the tumor, there is a new approach that exploits the specific character of tumors. In this design, cells are engineered to respond to hypoxia through the constitutively expressed transcription factor, hypoxia-inducible factor-1 alpha (HIF1α).^116^ A first approach implemented CARs fused with an oxygen-dependent degradation domain (ODD) of HIF1α.^117^ Under conditions of normoxia, the ODD becomes ubiquitinated, making the CAR protein proteasomal degrade. Although a CAR-ODD endowed CAR T cells with an improved ability to kill tumor cells under hypoxic conditions, the authors observed residual tumor cell killing under normoxic conditions. The second generation of this approach was developed as a dual oxygen-sensing method (Fig. 11A).^118–120^ This was achieved by fusing an ODD to the CAR as well as modifying the CAR's promoter to include hypoxia-responsive elements (HREs), which allowed HIF1α-mediated transcription of the CAR. An oxygen-sensing switch provides stringent hypoxia-dependent regulation of a CAR (Fig. 11B and C). Hypoxia-inducible CAR-T cells showed tumor-selective CAR expression and anti-tumor efficacy in various tumor models. The hypoxia-sensitive transcription switch significantly decreased CRS, off-tumor activation, and organ damage markers.^120^ Kosti *et al.* claim that patient populations should be selected by HRE-related biomarker expression.^119^ Because hypoxia is a commonly conversed character across cancer types, this approach is promising to provide a strategy to improve the safety of CAR-T cell treatments that fail due to toxicity.

## 8. Discussion

Researchers have been trying to control and ameliorate the toxicity of CAR-T cell therapies by testing diverse approaches to address unmet medical needs in the clinic. Each approach has different characteristics and strengths.

So far, there have been several strategies preclinically and clinically investigated for evolving CAR designs for self-termination to resolve CRS. iCasp9 has also been incorporated into CAR-T cells in clinical trials (NCT02414269), during which the inducer drug AP1903 activates iCasp9 dimerization to elicit CAR-T apoptosis during CRS. Moreover, surface labeling of CAR-T cells by CD20 or tEGFR has been proposed to eliminate CAR-T cells *via* CD20 or EGFR-targeted antibodies during severe CRS (NCT03618381; NCT03085173). Thus, kill switches display the potential for mitigating the effects of unintended CAR-T cell activation. However, these strategies rely on the irreversible elimination of CAR-T cells to reduce CRS, which is likely to simultaneously impair therapeutic efficacy.

On the other hand, CAR adaptor systems and transient CAR suppression are examples of techniques that can reversibly restrain CAR function, a feature which can yield greater flexibility and control than irreversible kill switches. These techniques can safeguard against toxicities other than CRS, including on-target, off-tumor responses, in which CAR-T cells lyse healthy, non-malignant cells that present the same target antigen as tumor cells. However, CAR-based adaptor systems act as ON switches for anti-tumor activity based on the presence of TAAs and adaptors themselves. This behavior differs from that of kill switches and transient CAR suppression, which serves as OFF switches upon the introduction of depleting/degrading agents. Fundamentally, an OFF switch for CAR-T cells would be riskier than an ON switch, as excess doses of external chemicals would be required to completely halt CAR-T cell activity. In return, OFF-switch CAR-T cells would not necessitate continuous support for *in vivo* persistence, in contrast to ON-switch CAR-T cells. Regardless of the type of system, the immediate effectivity (tocilizumab can reportedly resolve fever and other symptoms within hours^15,121^), efficiency and safety of drugs that control the activity of CAR-T cells must be carefully investigated before clinical application.

Hypoxia-sensing CARs have a distinct character compared to other CAR regulatory systems, in that their activity is automatically controlled based on the surrounding environment. As Kosti *et al.* mentioned, it will be important to verify whether unwanted activation occurs in noncancerous hypoxic conditions such as intestinal mucosa and ischemia, and to what extent hypoxia-sensing works in human cancers. Multiple clinical trials are underway to test the effectiveness of the adapter-based ON-switch CAR-T cells and have been summarized elsewhere.^122^ As dasatinib can be applied to virtually any CAR-T cells regardless of target antigens in theory, the results of the ongoing clinical trial (NCT04603872) are eagerly awaited to reveal the clinical practicality of the OFF-switch strategy.

The orthogonal IL-2/R system has tremendous potential for improving specificity for CAR-T cell therapy. The overall function of this cytokine engineering approach bears resemblance to the adaptor-based strategies designed to increase CAR-T cell safety. For example, like the adaptors, the dosage of orthogonal IL-2 determined both efficacy and toxicity of the CAR-T cells. A low dosage of 1 μg and a high dosage of 10 μg of STK-009 were evaluated, both with strong anti-tumor activity and the high dose yielding mild weight loss in mice.^66^ Furthermore, like the study conducted by Lee *et al.*,^45^ rigorously testing administration schedules (interruptions, frequency, *etc.*) could provide more data that informs scientists and clinicians about the safest and most effective practices to follow when overseeing the therapy in action. As a measure for additional control over safety, ortho-IL-2Rβ^+^ CAR-T cells could be further engineered with a knockout of WT IL-2Rβ. This would prevent the uncontrollable expansion of CAR-T cells due to WT IL-2-derived from the CAR-T cell itself or endogenous immune cells in patients. The requirement of this additional engineering might be revealed by *in vivo* studies of ortho-IL-2Rβ^+^ CAR-T cells in immunocompetent models in the future.

Considering their key role in CAR-T cell-related CRS, targeting macrophages would be a promising approach for increasing therapeutic safety. Drug repositioning of MTR can potentially facilitate smooth clinical translation of this approach. Practically, this approach would be used in combination with another strategy that would directly address CAR-T cells’ toxicity, as macrophage regulation will likely not be able to prevent toxicities that are not related to the macrophages themselves, such as on-target, off-tumor toxicity.

Apart from targeting macrophages or CAR-T cells directly, cytokine neutralization can pave the way for addressing the detrimental effects of unbridled cytokine diffusion. Self-neutralization of pro-inflammatory CRS cytokines (*e.g.* IL-6) offers the preferable option of automatically decreasing such cytokines’ concentrations, while avoiding immune-related damage. Future studies are needed to determine whether secreted or receptor-type neutralizers are preferable. Future clinical studies will uncover the advantages and disadvantages of cytokine self-neutralization CAR-T cells over separate injections of cytokine neutralizing antibodies. The importance of strategies that inhibit potentially detrimental cytokines derived from endogenous immune cells will certainly grow in the future as solid tumor therapies are increasingly being designed to actively engage host immune cells, such as armored T cells that secrete immune-modulatory proteins.^123–126^

## 9. Conclusion

An implicitly accepted feature of conventional CAR-T cell therapy is its propensity to develop CRS for the patient. As strategies evolve to eradicate advanced cancers, larger tumor burdens can induce greater cytokine production, increasing the likelihood of CRS. Despite its importance, the toxicity of CAR-T cells was sometimes overlooked during the development. Therefore, future CAR-T cell therapies should be designed smart, including features that constantly control cytokine release to avoid systemic damage to the patient. Molecular and cellular engineering based on immuno-oncology is crucial for finding solutions to maximize efficacy and minimize toxicity. Since each of the aforementioned strategies harness different properties to counteract CRS, they ought to be used in optimal combinations when attacking the TME. With the continued development and spread of molecular engineering technologies, clinical translation of more effective and safer adoptive immunotherapy will be feasible in the future.

## Conflicts of interest

Dr Ishihara is a co-founder and shareholder of ArrowImmune Inc., which develops anti-cancer immunotherapies. The other authors declare no conflicts of interest.

## Supplementary Material
